# Artificial Intelligence in Drug-Coated Cardiovascular Devices: A Narrative Review

**DOI:** 10.31083/RCM40892

**Published:** 2025-11-18

**Authors:** Rasit Dinc

**Affiliations:** ^1^INVAMED Medical Innovation Institute, New York, NY 10007, USA

**Keywords:** artificial intelligence, drug-coated cardiovascular devices, cardiovascular intervention, monitoring

## Abstract

Drug-coated cardiovascular devices (DCCDs), including drug-eluting stents (DESs) and drug-coated balloons (DCBs), have significantly advanced interventional cardiology by reducing restenosis and improving long-term outcomes. However, their effectiveness is limited by challenges such as patient-device mismatch, variability in drug delivery kinetics, and dependence on operator experience. Traditional strategies for device selection and performance evaluation are often inadequate to address patient-specific complexities. This narrative review aims to explore how artificial intelligence (AI) can improve the design, deployment, and monitoring of DCCDs, focusing on personalized treatment strategies, regulatory implications, and future innovations in interventional cardiology. A targeted literature search was conducted in PubMed, Scopus, and Web of Science between 2020 and 2025 using keywords such as “artificial intelligence”, “drug-eluting stents”, “cardiovascular devices”, “machine learning”, and “intravascular imaging”. Studies were included based on their relevance to AI applications in DCCD design, procedural support, or post-procedural monitoring. AI has demonstrated significant potential throughout the DCCD lifecycle. In design, machine learning models enable optimization of drug release kinetics and device geometry. During procedures, AI improves real-time intravascular imaging interpretation and provides guidance for precise device placement. Post-intervention, predictive analyses using patient data can aid in the early detection of complications such as in-stent restenosis. Furthermore, technical, regulatory, and ethical challenges remain, including model validation, data bias, and the need for transparency in decision-making algorithms. AI-driven approaches offer a promising paradigm for advancing cardiovascular device technology toward more adaptable, personalized, and efficient care. Integrating explainable, clinically validated AI systems with DCCDs can improve outcomes, reduce procedural variability, and support value-based care. Future research should prioritize real-time intraoperative feedback systems, adaptive AI models based on longitudinal patient data, and regulatory compliance and fairness strategies.

## 1. Introduction 

Cardiovascular diseases (CVDs) remain the leading cause of death and disability globally, accounting for and estimated 17.9 million deaths annually [1]. These not only make cardiovascular diseases the leading cause of death worldwide but also impose a significant economic burden, expected to rise from around US$400 billion annually to approximately US$1500 billion by 2050. This growing burden is fueled by both an aging population and the increasing prevalence of comorbidities such as diabetes, hypertension, and dyslipidemia [2]. These factors necessitate personalized and effective treatment approaches, particularly in the field of interventional cardiology.

Advances in interventional modalities over the past two decades, particularly the development of drug-coated cardiovascular devices (DCCDs) such as drug-eluting stents (DESs) and drug-coated balloons (DCBs), have significantly improved patient outcomes by reducing complications and the need for repeated revascularization procedures [3, 4]. However, current DCCD technologies still face significant limitations, including long-term risk of in-stent restenosis (ISR), patient-device incompatibility, and variability in drug release kinetics [5, 6]. Optimizing device performance remains inconsistent, with significant patient response variability affecting outcomes [7]. Current approaches to device selection and deployment rely heavily on operator experience and standardized protocols that fail to account for individual patient characteristics, lesion complexity, or anatomic variations [7, 8]. These challenges limit procedural precision and necessitate repeated interventions in a significant proportion of patients. Moreover, traditional risk stratification models often fail to capture the full spectrum of patient-specific variables that influence procedural success and long-term outcomes.

Artificial intelligence (AI) has the potential to transform the healthcare industry, including the cardiovascular domain [9]. Machine learning (ML) and deep learning (DL) within AI offer unprecedented capabilities in data analysis, pattern recognition, and predictive modeling that can optimize treatment selection, predict adverse events, and enhance procedural techniques. AI can be utilized to improve the design, deployment, and post-procedure evaluation of DCCDs [10]. AI’s ability to process and analyze vast amounts of heterogeneous data, including imaging, genetic, clinical, and procedural parameters, offers unique opportunities for developing personalized treatment plans based on individual patient characteristics and risk profiles [11].

While previous reviews have generally generalized the use of AI in cardiology without addressing the device-specific effects or regulatory challenges associated with AI-integrated cardiovascular implants, recent advances in quantum machine learning approaches have offered new paradigms for processing larger-scale medical datasets. These approaches have not yet addressed the application of AI in interventional cardiology workflows or implantable device innovation. However, quantum machine learning (QML)-based strategies, including cloud-based ones, have further expanded computational capabilities for complex cardiovascular disease classification and risk prediction, providing valuable insights, particularly in diagnostic modeling [12, 13].

The integration of AI with DCCDs spans the entire device lifecycle, from initial design and development to real-time procedural guidance and long-term outcome monitoring. AI algorithms can assist with pre-procedural planning for optimal device selection and placement strategies, provide real-time guidance during procedures to increase precision and reduce complications, and enable post-procedural monitoring for early detection of adverse events such as in-stent restenosis or thrombosis [14, 15, 16]. However, to our knowledge, there is currently no comprehensive review integrating these AI innovations into the lifecycle of DCCDs, from design and regulatory modeling to intraprocedural guidance and postprocedural surveillance. This gap limits the broader understanding of how AI can transform the future of smart cardiovascular implants.

However, ensuring the fairness, transparency, and clinical credibility of AI models remains a pressing concern. Furthermore, the integration of AI into clinical practice presents significant regulatory and implementation challenges. Current regulatory frameworks for AI applications in healthcare are still in their early stage, lacking a global standardization [17]. Regulatory agencies in the United States (US), the European Union (EU) countries, and other regions are working to create frameworks that balance the transformative capabilities of AI with patient safety and ethical considerations [18, 19, 20]. The complexity of validating AI-enabled medical devices, particularly those that learn and adapt over time, requires new approaches to clinical testing and post-market surveillance [21].

Therefore, this narrative review aims to fill this gap by analyzing and categorizing the existing literature on AI applications in DCCDs, from imaging-guided procedural support to AI-assisted drug administration modeling. We also highlight technical, regulatory, and ethical barriers to implementation and suggest future directions aligned with precision interventional cardiology.

### 1.1 Literature Review and Inclusion Criteria

This article used a targeted literature search using keywords such as “artificial intelligence”, “cardiovascular stents”, “drug-coated balloons”, “computational modeling”, and “intravascular imaging” through PubMed, Scopus, and Web of Science databases between 2020 and 2025. Peer-reviewed studies reporting original data, validated platforms, or ready-to-implement tools were emphasized. Studies were included based on relevance, originality, and clinical utility to AI applications in DCCDs. Reviews, preclinical studies, and conference proceedings were excluded unless they presented novel, validated models or datasets.

The key contributions of this comprehensive narrative review are as follows: (i) summarizing the clinical development and current limitations of DCCDs, including DESs and DCBs, to demonstrate the need for AI integration, (ii) systematically examining the latest AI models applicable to device design, intravascular imaging, and post-procedural monitoring, (iii) exploring how AI contributes to the development of novel DCCDs, including predictive drug delivery modeling and stent optimization, (iv) discussing the evaluation of the regulatory, technical, and ethical issues involved in translating AI-integrated DCCDs into clinical use, (v) identifying research gaps and suggesting future directions, such as adaptive AI tools for real-time procedural feedback.

### 1.2 Article Organization

The rest of this article is organized as follows: Section 2 provides background information on the current state of drug-coated cardiovascular devices, setting the clinical context for AI applications. Section 3 examines the role of AI in cardiovascular interventions by addressing pre-procedure planning, real-time guidance, and post-procedure monitoring. Section 4 focuses on AI applications in DCCD development, including device design optimization and drug release kinetics modeling. Section 5 discusses the various challenges associated with AI applications, including ethical, economic, and regulatory considerations. Section 6 acknowledges the limitations of this review, and Section 7 concludes with future perspectives and actionable recommendations for advancing the field.

## 2. Current Landscape of Drug-Coated Cardiovascular Devices

DCCDs have revolutionized the interventional cardiovascular field by combining localized drug delivery and mechanical support to diseased vessels, providing significant improvements in reducing restenosis rates and improving revascularization outcomes compared to bare-metal stents (BMSs) and plain balloon angioplasty (PBA) [3, 4]. This section provides essential background on the current DCCD landscape to establish the clinical context for AI applications.

### 2.1 Device Categories and Clinical Applications

#### 2.1.1 Drug-Eluting Stents (DESs)

DESs, metallic scaffold coated with antiproliferative drugs, represent the most widely used DCCDs in clinical practice [5]. The transition from first-generation devices using durable polymers containing sirolimus or paclitaxel to today’s biocompatible and bioresorbable platforms reflects ongoing efforts to balance efficacy against restenosis with improved safety profiles [22, 23]. Modern DES platforms combine advanced polymer technologies and new-generation limus family drugs (everolimus, zotarolimus) to optimize drug release kinetics and minimize long-term complications [7].

#### 2.1.2 Drug-Coated Balloons (DCBs)

DCBs deliver antiproliferative agents, most often paclitaxel, directly to the vessel wall during balloon angioplasty without permanent implantation. This offers particular advantages in in-stent restenosis, small vessel disease, and complex lesion subgroups where permanent hardware retention may be suboptimal [3, 24, 25].

### 2.2 Therapeutic Agents and Mechanisms

Current DCCD platforms primarily utilize two drug classes: the limus family of compounds (sirolimus, everolimus, zotarolimus), which inhibit mammalian target of rapamycin (mTOR) signaling and prevent smooth muscle cell proliferation, and paclitaxel, which disrupts microtubule dynamics to inhibit cell division and migration [26, 27, 28]. Emerging therapeutic approaches are exploring anti-inflammatory agents such as tacrolimus and corticosteroids to address the inflammatory components of restenosis [29, 30].

### 2.3 Key Clinical Challenges and Unmet Needs

Despite significant technological advances, there are several challenges that still require intelligence-driven solutions.

The first challenge is related to optimizing device performance. Current device selection is primarily based on angiographic measurements and operator judgment, potentially missing opportunities for patient-specific optimization based on lesion characteristics, vascular biology, and individual risk factors [8].

Another challenge is risk of ISR, which affects 5–15% of the patients with modern DES. This variability reflects the complex interactions between patient factors (genetic polymorphisms, diabetes, inflammatory status), lesion characteristics (calcification, vessel size, plaque composition), and device characteristics (polymer biocompatibility, drug release kinetics, and support design) [8, 31]. Late stent thrombosis (LST), while rare, remains a significant concern, necessitating prolonged antiplatelet therapy and careful patient evaluation [7]. Achieving optimal drug concentrations in target tissues while minimizing systemic exposure remains challenging, particularly in calcified lesions or complex anatomies where drug penetration may be compromised [6, 32].

Recent innovations have explored dual-drug delivery systems (e.g., paclitaxel-sirolimus combinations), polymer-free coatings, and feedback-controlled pharmacokinetics to enhance vascular healing while minimizing systemic exposure [32, 33, 34]. However, the widespread clinical adoption of these advanced systems is limited by the lack of patient-specific optimization and real-time decision support, creating an area where artificial intelligence has transformative potential.

### 2.4 The AI Opportunity

The complexity of optimizing DCCD performance across diverse patient populations and clinical scenarios creates an ideal environment for AI-driven innovation. The multidimensional nature of cardiovascular interventions, encompassing imaging data, procedural variables, patient characteristics, and device features, aligns with AI’s strengths in pattern recognition, predictive modeling, and decision support.

Modern interventional cardiology generates vast datasets through intravascular imaging [intravascular ultrasound (IVUS) and optical coherence tomography (OCT)], physiological assessments [instantaneous wave free ratio (iFR), fractional flow reserve (FFR)], angiographic analysis, and electronic health records [35]. These rich data sources, combined with evolving computational capabilities, enable AI applications that can improve every aspect of DCCD use, from initial device design to long-term patient follow-up.

The following sections examine how AI technologies are being integrated throughout the DCCD lifecycle to address these persistent challenges and unlock new possibilities for personalized cardiovascular care.

## 3. Role of AI Role in Cardiovascular Interventions

### 3.1 Technical Foundation and AI Methodologies

AI encompasses multiple computational approaches that have demonstrated significant potential in cardiovascular medicine [9]. The technical foundation of AI applications in DCCDs rests on several core methodologies, each optimized for specific clinical tasks.

Among AI methodologies, ML and its subfields are central to cardiovascular device applications [36]. Supervised learning approaches, including support vector machines (SVM), random forests, and gradient boosting methods (XGBoost, LightGBM), excel at risk prediction and outcome classification tasks. These algorithms achieve superior performance in predicting in-stent restenosis (area under the curve (AUC) 0.82–0.89) and bleeding complications (AUC 0.76–0.84) compared to traditional clinical scoring systems [32, 37].

DL, a subfield of machine learning based on artificial neural networks, has shown remarkable potential in processing high-dimensional imaging data. Convolutional neural networks (CNNs), particularly U-Net and ResNet architectures, are particularly effective in analyzing IVUS, OCT, and coronary computed tomography angiography (CCTA) and can automate plaque characterization, lumen measurement, and stent sizing [35]. For intravascular imaging, CNN-based segmentation algorithms achieve 92–95% accuracy in vessel boundary detection and 88–92% accuracy in plaque characterization [38, 39]. Recurrent neural networks (RNNs) and Long Short-Term Memory (LSTM) networks provide excellent results in temporal data analysis, enabling real-time monitoring of operational parameters and outcome prediction.

Reinforcement learning (RL), which learns optimal strategies through reward-based feedback, is emerging in interventional cardiology for procedural planning, such as selecting stent type and length based on lesion characteristics and simulated outcomes. Unsupervised learning techniques, including clustering and dimensionality reduction algorithms, are used to discover new patient subtypes or treatment response profiles from electronic health records and registries [40].

Computer vision (CV)-based advanced image processing techniques such as semantic segmentation, object detection (YOLO, R-CNN), and image registration algorithms enable automated analysis of angiographic and intravascular imaging data. These methods provide human-level performance in stenosis detection (sensitivity 94%, specificity 91%) and vessel measurement accuracy within 0.1 mm of expert judgment [35].

Natural language processing (NLP) techniques, including transformer-based models (BERT, GPT variants), facilitate the extraction of clinical insights from unstructured electronic health records, transactional reports, and literature analysis, supporting clinical decision-making and research synthesis [41].

AI systems in cardiovascular interventions are evaluated using established metrics [42, 43]. Important metrics for classification tasks include area AUC, sensitivity, specificity, and positive/negative predictive values. Mean absolute error (MAE), root mean square error (RMSE), and R-squared values are prominent in regression tasks. Apart from these, metrics such as the dice similarity coefficient and Hausdorff distance used for image analysis; C-index, net reclassification improvement (NRI), and integrated discrimination improvement (IDI) for survival analysis used in clinical validation are widely used.

AI technologies are increasingly being adopted in cardiovascular medicine to support diagnostic and procedural decision-making [9]. Broadly, AI simulates human cognitive functions, including learning, reasoning, and problem solving, using various algorithmic approaches. A key subset of AI is ML, which enables systems to learn patterns from data without the need for explicit programming. DL, a branch of ML, uses multilayered neural networks to model complex relationships in big data. DL has demonstrated particularly strong performance in cardiovascular imaging tasks due to its ability to automate feature extraction and improve classification accuracy [44].

NLP is increasingly being used to extract meaningful clinical information from unstructured data such as electronic health records or research literature. CNNs, a type of DL architecture, have proven particularly effective for image-based tasks and are widely used in computer vision, including automated analysis of coronary angiograms and IVUS [38, 39].

These innovations have led to the development of AI-integrated intravascular imaging platforms that support vessel segmentation, lesion detection, and real-time device selection. These tools provide rapid and standardized imaging interpretations, reducing operator variability and improving processing precision. For example, many commercially available AI tools now offer automatic vessel size estimation and lesion characterization, enabling personalized stent selection and optimization of percutaneous coronary intervention (PCI) strategies [10, 11].

Among the leading commercial platforms, the AVVIGO+ Multimodality Guidance System (Boston Scientific) exemplifies the integration of AI into intravascular imaging. It combines high-resolution IVUS with physiological data and uses a machine learning algorithm based on U-Net CNNs for automatic segmentation of the vessel lumen and its boundaries. A distinctive feature of the AI-powered AVVIGO+ system is the ability to automatically determine lesion length, proximal and distal reference segments, and the location of the minimal lumen area in the longitudinal view. This capability can significantly improve accurate and rapid device sizing without manual input. In a study by Matsumura *et al*. [35], this automation achieved 92.4% agreement with expert-based sizing for balloon selection, highlighting its clinical utility and sensitivity.

Another leading platform, the Ultreon 2.0 software (Version: 2.0, Abbott Vascular, Santa Clara, CA, USA), applies AI to OCT imaging. Ultreon 2.0 provides automatic detection and quantification of calcified plaque, providing key measurements such as total calcium arc, maximum thickness, and plaque length, which are necessary to assess the need for calcium modification before stenting. The system also detects the external elastic lamina (EEL) to assist with stent sizing and landing site selection. By automating these complex assessments, Ultreon 2.0 improves pre- and post-stenting decisions, increases the reproducibility of image interpretation, and reduces reliance on operator expertise [45]. Table 1 summarizes the key features of AI-enabled intravascular imaging software from selected commercially available examples.

**Table 1.** **Selected examples of commercially available AI in intravascular imaging and percutaneous intervention**.

AI-enabled tool	Company	Modality	AI capability	Clinical implication
AVVIGO+ Multimodality Guidance System, Version: Not specified	Boston Scientific, Marlborough, MA, USA	IVUS	Lumen and vessel segmentation, plaque burden detection, length measurement	Stent/balloon sizing, lesion length assessment, landing site selection, post-PCI optimization
Ultreon 2.0, Version: 2.0	Abbott Vascular, Santa Clara, CA, USA	OCT	EEL detection, calcium arc/thickness/length recognition	Stent/balloon sizing, calcium modification planning
Gentuity HF-OCT, Version: Not specified	Gentuity LLC, Sudbury, MA, USA (distributed by Nipro Corporation, Osaka, Japan)	HF-OCT	Lumen and MLA/MSA detection, segmentation, guidance for catheter identification	PCI decision support, extension monitoring, image optimization
HyperVue, Version: Not specified	SpectraWAVE, Bedford, MA, USA	NIRS-OCT	Lumen/EEL detection, calcium and lipid core detection, stent evaluation	High-risk plaque detection, advanced calcium therapy, post-PCI optimization
CathWorks FFR Angio, Version: Fourth generation	CathWorks, Kfar Saba, Israel	Angiography	FFR mapping from angiograms	Lesion-specific FFR guidance for PCI
CAAS IntraVascular, Version: 2.0	Pie Medical, Maastricht, Netherlands	Angiography, OCT, IVUS	Automatic segmentation and coronary tree reconstruction	Stent placement assessment and placement support
IntraSight Imaging, Version: IntraSight 7	Philips, Amsterdam, Netherlands	IVUS, iFR, FFR	Real-time plaque/lesion detection, vessel wall analysis	iFR/FFR decision making, precise stent placement
QAngio XA, Version: 7.3	Medis Medical Imaging, Leiden, Netherlands	Angiography	3D reconstruction and stenosis quantification	Stenosis assessment, PCI planning

Abbreviations: AI, artificial intelligence; IVUS, intravascular ultrasound; PCI, percutaneous coronary intervention; OCT, optical coherence tomography; HF, high frequency; NIRS, near-infrared spectroscopy; iFR, instantaneous wave-free ratio; FFR, fractional flow reserve; CAAS, Cardiac analysis system; EEL, external elastic lamina; MLA, minimal lumen area; MSA, minimal stent area.

Taken together, these AI technologies underpin current and emerging applications in the development and implementation of DCCD. The following sections discuss how these tools support planning, real-time intervention, post-procedure prediction, and device design.

### 3.2 AI-Supported Pre-Procedural Planning

#### 3.2.1 Clinical Decision Support Systems (CDSSs)

AI technologies transform preprocedural planning by integrating multimodal data sources to optimize patient selection and procedural strategies. ML models that analyze demographic, clinical, and imaging parameters achieve superior performance in identifying patients who will benefit from intervention compared to medical therapy (AUC 0.87 vs. 0.72 for traditional risk scores).

DL algorithms, particularly 3D CNN architectures, automates coronary artery segmentation and stenosis quantification from computed tomography angiography (CTA) datasets. Advanced models, including ResNet-50 and DenseNet architectures, achieve 94% accuracy in detecting significant stenosis (>70% diameter reduction) and provide automated calcium scoring with correlation coefficients >0.95 compared to manual analysis [46, 47].

Physics-informed neural networks (PINNs) enable noninvasive fractional flow reserve calculation by solving the Navier-Stokes equations using patient-specific geometric models. These approaches provide diagnostic accuracy comparable to invasive FFR (AUC 0.93, sensitivity 90%, specificity 91%) while reducing procedure complexity and radiation exposure [48].

AI-enabled simulation platforms use finite element analysis combined with machine learning to predict procedure outcomes. These systems model stent deployment mechanics, drug delivery patterns, and vascular response, enabling virtual testing of different device strategies before intervention [15, 16].

#### 3.2.2 Operator Training and Skill Development

Although AI has enabled significant improvements in procedural decision-making, real-world implementation is highly dependent on operator expertise, particularly in anatomically complex interventions. AI tools are now being integrated not only to improve clinical planning but also to reduce inter-operator variability and support personalized training environments.

Patient-specific digital twins created using AI-powered computational modeling provide realistic training environments for interventional procedures. These virtual replicas include real-time physics simulation, haptic feedback, and adaptive learning algorithms that adjust difficulty based on trainee performance metrics [49].

Augmented reality (AR, allowing visualization of data and virtual objects in a physical environment) and virtual reality (VR, creating an immersive virtual environment that allows interaction with virtual objects and scenarios as if they were real), combined with AI-powered analytics, have opened up new possibilities for training interventionalists and improving their procedural skills [49]. For example, VCSim3 (Mentice AB), a real-time virtual reality simulation software, creates a realistic and dynamic training environment by simulating the physical behavior of catheters and guidewires, helping healthcare trainees effectively practice procedural techniques. ML algorithms analyze trainee performance patterns to identify skill gaps and provide personalized feedback to accelerate learning curves [50]. These tools allow medical students, residents, and other healthcare professionals with limited experience to develop a deeper understanding and clinical skills in vascular interventions.

AI systems monitor procedural metrics (catheter manipulation time, contrast agent use, radiation exposure) and correlate these with outcomes to determine the most appropriate procedural techniques. Reinforcement learning algorithms can recommend procedural changes in real time based on accumulated experience data [51].

### 3.3 AI in Real-Time Guidance

AI technologies have played a significant role in improving real-time decision-making during PCI by assisting operators with dynamic image interpretation, procedural navigation, and device positioning. Modern AI-enabled intravascular imaging platforms support intraprocedural adjustments and optimize procedural outcomes in real time.

#### 3.3.1 AI-Supported Intravascular Imaging Platforms

**AVVIGO+ system (Boston Scientific)**: This AI platforms utilizes U-Net CNN architectures for automated IVUS image segmentation. The system provides real-time measurements of minimum lumen area (MLA), vessel diameter, and lesion length, achieving 92.4% agreement with expert vessel sizing. Machine learning algorithms automatically determine optimal landing zones and recommend appropriate device sizes, reducing processing time by 15–20% [35].

**Ultreon 2.0 platforms (Abbott Vascular)**: Advanced OCT analysis using deep learning enables automated calcium detection and quantification. The system’s CNN-based algorithms achieve 89% accuracy in calcium arc measurement and 91% accuracy in thickness assessment, providing critical data for pre-stent optimization strategies. External elastic lamina detection algorithms demonstrate 94% correlation with expert annotations [45, 52].

Among these platforms, Gentuity High-Frequency (HF)-OCT (Nipro) can automate recognition of the MLA, minimal stent area (MSA), and external elastic lamina (EEL), improving stent sizing and landing zone decisions, reducing operator subjectivity and the risk of restenosis or stent failure [53]. Platforms such as IntraSight (Philips) and CAAS IntraVascular (Pie Medical) provide further support to operators by providing real-time feedback during stent deployment, leveraging automated segmentation and plaque characterization across IVUS, OCT, and angiography modalities. These capabilities transform procedural guidance into a dynamic, data-driven process that improves outcomes and minimizes complications [10].

These platforms integrate multiple AI components, including real-time inference engines, automated image quantification, and predictive modeling for device selection [54, 55].

#### 3.3.2 Advanced Guidance Technologies

**Augmented reality (AR) systems**: These systems such as SentiAR (St. Louis, MO, USA) project holographic anatomical representations directly into the operator’s field of view. CV algorithms enable real-time registration of virtual objects with fluoroscopic images, improving spatial understanding and reducing procedural complexity [56].

**Robotic-assisted interventions**: With improved precision, fewer operative complications, and reduced occupational hazards, robotics has become increasingly prevalent in vascular interventions [57]. AI-assisted robotic systems such as CorPath GRX (Corindus Vascular Robotics) integrate machine learning algorithms for optimal catheter navigation. These systems reduce radiation exposure for operators by 95% while maintaining procedural success rates above 98%. RL algorithms refine navigation strategies by learning from procedural feedback to optimize catheter trajectories [58].

**Integration of physiological assessment**: AI-based functional assessment algorithms enable noninvasive FFR estimation from angiographic images, bypassing the need for pressure wire-based measurements in select cases. Deep learning models trained on paired angiographic-FFR datasets provide over 90% diagnostic accuracy (AUC 0.91) [59].

The integration of AI not only improves intraprocedural accuracy, but also improves operator and patient safety through optimized planning, decision support, and reduced radiation exposure. Moving forward, real-time AI integration is expected to evolve towards closed-loop systems that continuously learn from previous cases and adapt to operator preferences. The exploration of incorporating haptic feedback, haptic systems, and augmented reality (AR)-based visualization to improve procedural accuracy is ongoing. However, widespread clinical implementation will rely on prospective validation, regulatory compliance, and operator training.

### 3.4 AI in Postprocedural Monitoring and Outcome Prediction

#### 3.4.1 Predictive Analysis for Complication Prevention

Although still in the early stages, AI technologies are increasingly involved in the post-procedure phase of interventions with DCCDs. Post-procedural management includes monitoring complications such as ISR, thrombosis, and bleeding, and predicting long-term outcomes such as major adverse cardiovascular events (MACE). This enables proactive management strategies.

ML models has been trained in electronic health records (EHRs) to predict complications such as ISR and MACE. These models incorporate procedural variables, patient characteristics, and imaging parameters achieve superior predictive performance compared to traditional risk scores [31, 37]. Using variables such as stent type, lesion complexity, and comorbidities, XGBoost algorithms analyzing 47 clinical variables demonstrated an AUC of 0.89 for 12-month ISR prediction, compared to 0.67 for traditional scoring systems [36].

DL models analyzing intravascular imaging parameters, platelet function testing, and genetic markers achieve 85% accuracy in predicting stent thrombosis within 30 days. These systems provide personalized dual antiplatelet therapy duration recommendations, potentially reducing bleeding risk without increasing ischemic risk [60].

Ensemble learning approaches combining multiple algorithms (random forest, gradient boosting, neural networks) provide personalized bleeding risk assessments with c-statistics of 0.82–0.86 and significantly outperform traditional bleeding scores (c-statistics of 0.64–0.71) [61].

#### 3.4.2 Automated Monitoring Systems

Some trending applications are emerging in post-procedure follow-up [62]. One example is imaging-based longitudinal analysis. CNN-powered platforms automatically analyze follow-up angiography, IVUS, or OCT scans to measure lumen narrowing over time. These tools offer improved sensitivity compared to traditional monitoring methods, facilitating earlier detection of restenosis before clinical symptoms appear. Again, wearable device integration and remote monitoring tools can detect arrhythmic events or hemodynamic instability while monitoring physiological signals (e.g., electrocardiogram (ECG), activity monitors) post-PCI. NLP algorithms continuously monitor clinical notes, lab values, and imaging reports to identify early signs of complications.

**Fig. 1.**
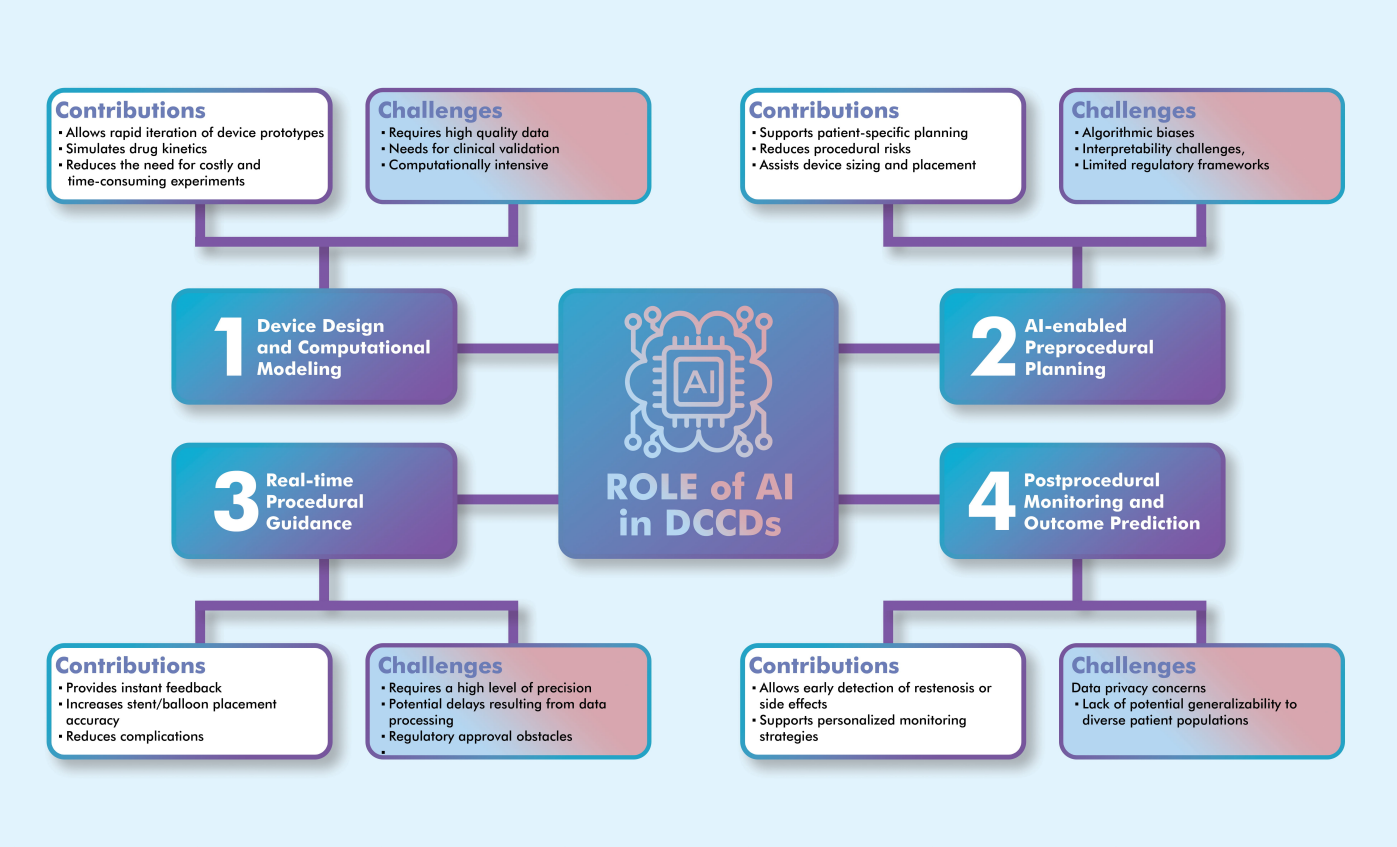
**AI applications across the lifecycle of drug-coated cardiovascular devices, showing the integration points, key technologies, and clinical outcomes at each stage**. The illustration was drawn using the Adobe Creative Suite Package [(Illustrator, version 28.7.1 and Photoshop, version 25.12) (Adobe Systems Incorporated, San Jose, CA, USA)].

In conclusion, AI-powered procedures make significant contributions to pre-intervention planning compared to traditional methods. They assist in predicting procedure duration, making decision in unforeseen circumstances, and optimizing surgical workflows. Furthermore, they can improve diagnostic and prognostic outcomes in the post-operative setting. The ability of AI to enhance risk stratification, facilitate complex imaging analyses, and support real-time clinical decision-making highlights its potential to improve patient outcomes and increase procedural efficiency. Fig. 1 illustrates the comprehensive integration of AI technologies across the entire cardiovascular intervention lifecycle, the interconnected nature of these applications, and their collective impact on clinical outcomes.

Benchmarks have shown that DL models often outperform traditional ML techniques on tasks requiring high-dimensional feature extraction. For example, CNNs trained on intravascular OCT data have achieved segmentation accuracies exceeding 90%, outperforming traditional support vector machines or random forest classifiers in both precision and recall [42]. However, while CNNs are superior for image-based inputs, gradient boosting models such as XGBoost demonstrate greater stability and interpretability on structured clinical datasets, particularly for risk stratification and dual antiplatelet therapy duration prediction [60]. These performance discrepancies underscore the importance of aligning model architecture with the data modality and clinical purpose. Despite impressive metrics (AUC >0.90 in many retrospective studies), concerns about generalizability persist, especially when models trained on single-center datasets are applied to populations with varying comorbidities and procedural standards.

## 4. AI in Drug-Coated Devices Development

The integration of AI into the DCCD development process represents a paradigm shift from traditional experimental approaches to data-driven, predictive design methodologies. By leveraging ML, DL, and RL, the entire DCCD lifecycle from concept to validation is being accelerated and refined.

Traditional development typically involves years of laboratory testing, animal experiments, and phased clinical trials. In contrast, in silico testing, supported by ML/DL and mechanistic modeling, enables virtual evaluation of device mechanics and drug delivery, supporting accelerated development cycles. Regulatory bodies such as the US FDA are increasingly recognizing the value of these virtual (*in silico*) clinical trials as a supplement or alternative to traditional clinical trials during regulatory approval processes [18, 63].

### 4.1 Device Design

The design and material composition of DCCDs significantly affect their performance. AI algorithms are widely used in stent and balloon design, particularly for optimizing structural geometry, mechanical behavior, and drug delivery efficiency. For example, supervised learning models trained on past stent deployment results have achieved over 90% predictive accuracy in predicting strut malposition and mechanical deformation [54]. Gaussian process regression models and Bayesian optimization techniques explore large design spaces to identify optimal configurations that balance mechanical performance, hemodynamic properties, and drug delivery characteristics. These approaches reduce design iteration cycles from months to weeks while simultaneously examining thousands of design variants [63, 64, 65].

ML and DL models are used to predict how design parameters (e.g., support geometry, polymer composition, drug-polymer interactions) affect device behavior. For example, supervised learning algorithms trained on clinical, and materials datasets have been used to optimize scaffold thickness and expansion profiles for novel stents [54, 64]. Samant *et al*. [66] demonstrated that AI-based modeling significantly improved the mechanical performance and drug distribution profile of everolimus-eluting stents specifically designed for left main coronary interventions. Similarly, Poletti *et al*. [64] developed an image-based, patient-specific digital twin approach to virtually model and test stent deployment strategies. These tools allow for iterative design testing under physiological conditions without physical prototyping, accelerating the development cycle and reducing costs.

In recent years, in silico studies based on finite element analysis (FEA) have been conducted to consider the geometric and material properties of intravascular devices such as stents and balloons, their different deployment strategies, and the patient’s vascular anatomical characteristics. FEA frameworks, when combined with RL, further support iterative modeling of intravascular mechanics under pulsating flow conditions. These frameworks enable rapid testing of structural changes and device positioning strategies without the need for physical prototyping [54, 64].

AI-driven computational modeling allows the simulation of various design parameters through in silico testing and enables virtual models to predict the physical and biological behavior of devices under various conditions. For example, stent designs can be tested in virtual environments for durability, hemodynamic performance, and drug release properties. This provides significant advantages in optimizing stent designs and accelerating the time from inception to clinical use [63]. RL techniques have also been applied to simulate catheter-based deployment strategies, providing adaptive guidance to minimize misplacement and vessel wall stress [54, 55]. Such simulation environments, when integrated with patient-specific imaging data, form the basis for AI-assisted design processes tailored to individualized anatomy and lesion morphology.

### 4.2 Computational Modeling for Drug Release Kinetics

Modeling drug diffusion and pharmacokinetics in complex vascular environments remains a fundamental challenge in DCCD development. By analyzing large datasets of material properties and clinical outcomes, AI can also help select optimal polymer coatings and drug combinations. This approach could significantly contribute to the development of DCCDs with improved biocompatibility and targeted drug delivery capabilities [32, 67].

Although DCCDs are widely used to reduce the rate of restenosis, ISR remains a challenge. The development of new DCCDs requires observation of the pharmacodynamic and pharmacokinetic properties of the loaded drugs, as well as testing for efficacy and safety. This process is time-consuming and costly [22, 68]. In the context of drug kinetic modeling, AI can make a powerful contribution to overcoming these limitations. AI can play an important role in optimizing various aspects, from drug release from the stent or balloon to drug transport in the blood stream and passage through the vascular wall. For example, AI-assisted algorithms can significantly contribute to the design of polymeric biocompatible cardiovascular devices with tunable mechanical and release properties, as well as optimize process parameters for coating technologies to achieve desired thickness, uniformity, and adhesion [69].

AI algorithms have enabled the modeling of complex pharmacokinetic and pharmacodynamic processes. For example, supervised CNNs have been trained on in vitro and animal model data to predict arterial wall drug concentration gradients with an average absolute error of less than 10% [69]. Graph neural networks (GNNs) have demonstrated potential for modeling drug diffusion pathways across non-uniform vascular tissue structures. Gracia’s computational framework modeled stent-based delivery systems using CNN-assisted simulations and validated outputs against physical measurements in porcine artery models [69]. These AI-assisted simulators accurately replicate *in vivo* behavior, reducing reliance on animal testing.

Beyond passive diffusion, AI models are now contributing to the design of feedback-controlled delivery systems that integrate biosensor inputs (e.g., endothelial response, flow shear stress). These systems can adapt drug release rates in real time using reinforcement learning controllers [33, 34].

AI models also play a key role in the development of patient-specific DCCDs. Vascular properties, such as calcification in the lesion composition, have a significant impact on drug absorption and distribution. This underscores the importance of modeling binding-diffusion kinetics and developing strategies for lesion-specific DCCDs [6]. AI algorithms are also transforming the personalization of antiplatelet therapy by customizing the dosage for each the patient. Traditional fixed dual antiplatelet therapy (DAPT) may not be optimal for all patients in balancing the risks of ischemia and bleeding. In this regard, ML models have been created to tailor the duration of DAPT to individual patient profiles [70]. Specific AI models such as XGBoost have been developed for this purpose [60]. These approaches hold the promise of personalizing not only device configurations but also pharmacotherapy regimens.

Table 2 summarizes the main types of AI models applied at various stages of the DCCD development process, including their core functions, advantages, and limitations.

**Table 2.** **Common ML models used in DCCD development tasks**.

Algorithm/Model	Application	Strengths	Limitations
Convolutional neural network (CNN), e.g., ResNet, U-Net	Drug diffusion modeling, image segmentation	High accuracy in image-based tasks	Large, labeled datasets are needed
Graph neural networks (GNNs)	Modeling vascular drug distribution	Captures complex topologies	Computationally intensive
Bayesian Optimization	Stent material and geometry design	Efficient search space exploration	Performance depends on surrogate model quality
Reinforcement Learning	Adaptive drug delivery control	Learns from dynamic feedback	Slower convergence; needs tuning
XGBoost	Personalized DAPT	High interpretability and speed	Can overfit small datasets

Abbreviations: ML, machine learning; DCCD, drug-coated cardiovascular devices; DAPT, dual antiplatelet therapy.

### 4.3 Integration With Imaging and Manufacturing

AI-powered integration of imaging modalities (e.g., OCT, IVUS, CT) into design workflows enables real-time feedback loops between anatomy, device design, and delivery simulations. Advanced segmentation tools based on U-Net and DenseNet architectures achieve Dice similarity coefficients >0.9 for vessel and lesion segmentation, enabling precise simulation of device-tissue interactions [35, 53] These imaging algorithm pipelines are essential for reducing geographic mismatch and customizing drug delivery profiles based on lesion morphology and calcification.

In the manufacturing arena, computer vision models, including generative adversarial networks (GANs), have been used to detect defects in polymer coatings and structural alignment. Real-time optical inspection systems have demonstrated over 95% accuracy in identifying microstructural inconsistencies, improving both quality control and manufacturing efficiency [42, 43].

**Fig. 2.**
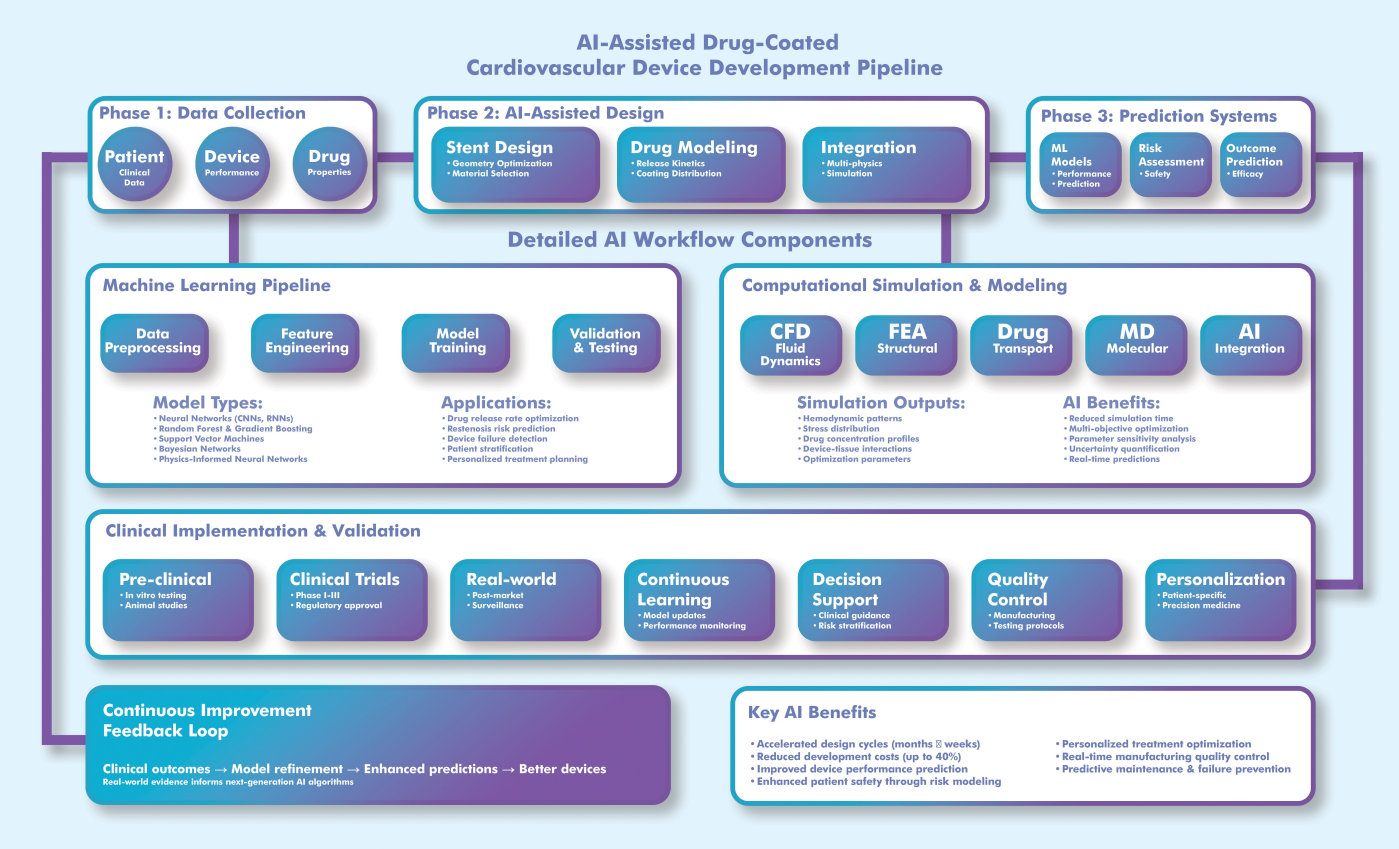
**Workflow for AI-assisted development of drug-coated cardiovascular devices (DCCDs)**. This process demonstrates the integration of AI algorithms into device design, drug release modeling, and virtual testing, highlighting feedback loops for optimization and regulatory validation. The illustration was drawn using the Adobe Creative Suite Package [(Illustrator, version 28.7.1 and Photoshop, version 25.12) (Adobe Systems Incorporated, San Jose, CA, USA)]. CFD, computational fluid dynamics; FEA, finite element analysis; MD, molecular dynamics.

To improve the interpretability of complex workflows, Fig. 2 summarizes the AI-assisted pipeline for DCCD development, including design optimization, pharmacokinetic modeling, and simulation-guided evaluation.

## 5. Challenges of AI in Drug-Coated Cardiovascular Devices Practice

While the integration of AI into DCCD applications is promising, it faces numerous technical, ethical, regulatory, and economic challenges. These challenges require systematic approaches and coordinated solutions for AI to realize its full potential in cardiovascular interventions.

### 5.1 Ethical Challenges and Mitigation Strategies

Applications of AI in healthcare, including its integration into cardiovascular interventions, raise several ethical challenges that need to be addressed. One of these challenges is data privacy and security. AI systems often rely on large datasets containing sensitive personal health information. Risks such as data breaches, unauthorized use, and cyberattacks can lead to discrimination or harm [71]. Regulatory bodies, such as the Health Insurance Portability and Accountability Act (HIPAA) in the United States and the General Data Protection Regulation (GDPR) in the European Union, are working to reduce these risks [72, 73]. For example, model inversion is a type of attack that exploits the final output of an AI model. It can reconstruct or extract training data from the outputs of a machine learning model, potentially exposing sensitive information. Attackers use the model’s behavior to “reverse engineer” the data it was trained on, even without direct access to the original dataset. These attacks pose significant privacy risks, especially when dealing with sensitive data like medical records or personal information [74]. Advanced privacy-preserving technologies offer robust solutions to these challenges. Differential privacy, which provides quantifiable privacy (ε ≤ 1) guarantees for cardiovascular risk models, can offer a mathematical framework for privacy protection [75].

Algorithmic bias and fairness are another important challenge. AI models trained on biased or non-diverse datasets often struggle when applied to populations different from those on which they were trained [76, 77]. Developing AI applications that address specific health needs at the local community or individual level can hinder the creation of robust AI models due to limited data availability or variability. However, a comprehensive bias reduction framework addresses these challenges through a variety of approaches. Bias-detection algorithms, such as fairness-aware machine learning and statistical equity tests, can accurately detect unfair model behavior. Regular fairness audit protocols using standardized fairness metrics ensure ongoing compliance with equity standards.

The third challenge is explainability and transparency. Many AI models, especially those using deep learning, operate as “black boxes”. Their opaque nature hinders trust and accountability. Transparent or federated AI systems and explainable AI (XAI) approaches are encouraged to increase user trust [65, 66]. Physicians must trust and understand algorithmic outputs, especially when considering factors affecting stent selection, drug-coating parameters, or treatment duration. Therefore, explainability should be a non-negotiable requirement for AI applications in interventional cardiology. XAI methods such as SHAP (SHapley Additive Explanations), LIME (Local Interpretable Model-Independent Explanations), and attention heatmaps are increasingly being used to clarify model rationale for individual predictions. These tools help uncover hidden biases and increase user confidence. However, considering the difficulties that explainability brings with it trade-offs in terms of model complexity, latency, and interpretation accuracy, hybrid strategies should not be ignored [39, 73, 78].

The fourth challenge is accountability and responsibility. Defining the roles among developers, clinicians, and institutions in AI-driven clinical decisions remains a challenge. Unfortunately, current legal systems have difficulty assigning responsibility for this issue [17]. Finally, global inequalities and accessibility to services can be addressed. AI tools may not be accessible to the desired level in low-resource environments due to reasons such as cost, infrastructure, or local barriers. Minimizing this requires adapting AI to local health needs and encouraging international regulatory cooperation [39, 79, 80].

Furthermore, traditional informed consent models are inadequate for continuously learning and evolving AI systems, creating ongoing consent challenges [81]. Blockchain-based systems and natural language processing systems could provide significant improvements [82, 83].

Techniques such as federative learning and adversarial bias removal have been proposed to increase data privacy and fairness in AI models for cardiovascular applications [17]. These methods can minimize demographic bias and improve generalizability.

### 5.2 Cost Aspect

Healthcare applications, including AI-based models, require initial setup, training, infrastructure, and other investments requirements. This not only presents a technical hurdle for AI implementation but also poses significant economic burdens. Economic impact metrics are critical in calculating these costs [84]. Traditional health economics models struggle to capture the dynamic benefits and costs of AI, especially for systems that improve over time [85]. Advanced economic modeling, such as dynamic cost-effectiveness models (e.g., Markov models), value-based pricing, total cost of ownership, and budget impact analysis, can address these challenges [84]. Integrating AI with these strategies has the potential to significantly reduce healthcare costs while improving diagnostic accuracy and treatment efficiency. For example, AI-assisted screening for diabetic retinopathy has been shown to lead to better health outcomes at lower costs than traditional methods [47]. Therefore, it is crucial to address the economic impact of AI adoption in healthcare.

On the other hand, the high initial costs of AI systems and the need for ongoing maintenance and updates pose significant implementation challenges. Furthermore, the lack of standardized methodologies makes it difficult to analyze the economics of AI interventions and understand their value. This challenge is particularly evident in regions with health inequalities [79, 86].

### 5.3 Validation Challenges

Rigorous validation of AI algorithms is critical for the development and clinical deployment of DCCDs. Validation strategies typically involve multistage testing. These include internal validation (e.g., cross-validation within the training set), external validation using independent datasets to assess generalizability, and prospective validation within clinical workflows [10, 16, 63].

Unlike static software tools, AI systems (especially those using deep learning) learn from large, high-dimensional datasets and can improve over time. Therefore, traditional validation metrics need to be expanded to assess both model performance and robustness under real-world variability. Performance metrics such as accuracy, AUC, sensitivity, specificity, and calibration should be tailored to each use case. Applications for DCCDs include predicting ISR, optimizing drug release kinetics, or customizing dual antiplatelet therapy duration [36, 60, 70].

Digital twin-based simulation frameworks and high-fidelity virtual experiments are emerging as complementary validation methods, reducing reliance on animal studies, and accelerating preclinical evaluation [64, 69]. These systems have the potential to play a critical role in modeling stent performance, drug elution dynamics, and vascular tissue interactions under physiological flow conditions.

### 5.4 Regulatory Considerations

It is crucial to establish validated and approved comprehensive regulatory frameworks to govern the application of these technologies. This is necessary to protect patients from misdiagnosis, misuse of personal data, and biases embedded in algorithms [87, 88]. Despite the intense efforts of regulatory bodies in some countries, there is still no global regulatory framework for AI applications in healthcare. Currently, the global regulatory environment primarily regulates the use of AI in healthcare for medical devices, specifically Software as a Medical Device (SaMD) [89]. Regulatory agencies such as the US Food and Drug Administration (FDA), the European Commission, and the UK Medicines and Healthcare products Regulatory Agency (MHRA) are developing frameworks to address the inherent risks of AI/ML-driven systems [18, 19, 88, 90].

The FDA’s Predetermined Change Control Plan (PCCP) framework is particularly important for machine learning-enabled devices, enabling post-approval algorithm updates while maintaining safety and performance assurance [18]. Similarly, the proposed EU Artificial Intelligence Act mandates risk stratification, documentation, and human oversight for high-risk AI applications in healthcare [19]. National organizations such as National institute for Health and Care Excellence (NICE (UK)) and the World Health Organization (WHO) have published evidence standards frameworks for AI tools that emphasize clinical effectiveness, economic value, and patient safety. These are crucial for deploying AI in DCCD personalization, where inaccurate predictions can have disastrous consequences [20, 80, 86].

AI/ML models continuously learn and improve over time, requiring adaptive algorithms and presenting unique regulatory challenges. To address this issue, the FDA introduced the “Proposed Regulatory Framework for Changes to AI/ML-based SaMD” in April 2019. This framework adopts a “total product life cycle (TPLC)” approach, acknowledging that AI/ML-based software frequently evolves as it learns from new data [90]. Regulatory fragmentation across different regions poses significant obstacles to global AI deployment. Harmonization initiatives such as the International Medical Device Regulators Forum (IMDRF) and ISO/IEC Standards (including ISO 14155 and ISO 13485) are addressing these challenges through various international efforts [91, 92].

Ensuring that AI algorithms perform reliably across diverse patient populations and clinical settings is essential. Regulators require robust clinical evidence for AI-based medical devices, but traditional clinical trial designs may be insufficient for adaptive AI systems [74]. Adaptive trials utilize Bayesian designs, allowing for protocol changes based on accumulated evidence and significantly increasing efficiency. Real-world evidence obtained through post-market surveillance using electronic health records and registry data enables continuous safety and efficacy monitoring. Digital biomarkers enable continuous monitoring using wearable devices and smartphone sensors, providing high-frequency outcome data [93, 94, 95].

## 6. Limitations

This narrative review offers a comprehensive overview of AI applications in DCCDs. However, it is important to acknowledge that there are several key limitations. Firstly, while a structured and targeted literature review was conducted using substantial databases and peer-reviewed sources, it does not adhere to the rigorous methodology of a systematic review. Therefore, there is a possibility of selection bias, and some relevant studies may have been inadvertently excluded. Secondly, the rapidly evolving nature of AI in healthcare means that some new technological developments or regulatory changes may not have yet been addressed at the time of writing.

Thirdly, although this review covers a wide range of topics, from device design and drug modeling to regulatory and ethical considerations, it does not provide a quantitative meta-analysis of outcomes associated with AI-assisted DCCDs. The performance of AI models often depends on data quality, population heterogeneity, and clinical validation status, which vary significantly across studies. Therefore, comparisons between studies should be interpreted with caution. Fourth, many studies cited here are based on early-stage data, simulated environments, or animal models. Translational gaps remain significant when considering generalizability to human populations or real-world intervention settings. Furthermore, a few of the AI platforms discussed have received regulatory approval or been prospectively validated in large-scale trials, limiting their immediate applicability.

Finally, this review emphasizes technological and algorithmic advancements rather than health economic modeling or interdisciplinary integration with behavioral, operational, or social determinants of cardiovascular health. These issues warrant future investigation to ensure the equitable and sustainable implementation of AI-enabled drug-coated cardiovascular technologies.

## 7. Future Perspective

The future integration of artificial intelligence (AI) into DCCDs must progress from theoretical promises to practical, patient-centered systems. One of the most urgent technical challenges is developing real-time intraoperative AI feedback systems. These systems can analyze intraprocedural imaging, vascular dynamics, and device positioning in real time to guide clinicians during stent or balloon deployment, reducing complications such as misplacement or incomplete lesion coverage.

Another crucial area is adaptive AI models that can learn from individual patient responses. By combining reinforcement learning and fusion learning architectures, these models can improve treatment recommendations, drug delivery strategies, and follow-up protocols over time. For example, DCCDs can regulate drug release kinetics based on evolving local hemodynamic signals or tissue responses.

To ensure the safe and effective implementation of these technologies, future research should prioritize explainability, generalizability across populations, and regulatory compliance. This involves using interpretable AI methods (e.g., SHAP, LIME) and validation frameworks that meet FDA and CE requirements for real-world clinical decision tools.

## 8. Conclusion

This review highlights the increasing role of AI in the design, deployment, and monitoring of DCCDs. Theoretically, AI represents a shift from empirical trial-and-error methods to predictive, personalized modeling. In practice, it enables enhanced personalization, device optimization, and procedural precision through advanced data integration and real-time decision-making.

This study provides several key contributions. These include: a comprehensive synthesis of AI applications across the DCCD lifecycle; a detailed examination of technical enablers, including drug delivery modeling and patient-specific stent simulation; identification of key challenges in model validation, interpretability, and ethical oversight; and practical recommendations for bridging the gaps between in silico tools and clinical implementation.

While AI show promise in enhancing cardiovascular device innovation, its clinical adoption faces obstacles such as biased training data, lack of external validation, algorithmic opacity, and fragmented regulatory standards. Overcoming these limitations requires a focused effort to integrate standardized assessment criteria, conduct multicenter studies, and establish ethical frameworks emphasizing fairness, transparency, and accountability.

Future research should concentrate on developing intraoperative AI guidance systems for real-time device optimization, implementing adaptive learning frameworks to personalize therapy over time, and establishing regulatory-compliant validation processes for AI-integrated DCCDs.

By advancing in these areas, AI-powered cardiovascular implants can move from innovation to clinical reality, bringing precision, safety, and personalization to interventional cardiology.
